# Prospective Study of Modifiable Risk Factors of Arterial Hypertension and Left Ventricular Hypertrophy in Pediatric Patients on Hemodialysis

**DOI:** 10.1016/j.ekir.2024.03.016

**Published:** 2024-03-18

**Authors:** Dagmara Borzych-Dużałka, Rukshana Shroff, Bruno Ranchin, Yihui Zhai, Fabio Paglialonga, Jameela A. Kari, Yo H. Ahn, Hazem S. Awad, Reyner Loza, Nakysa Hooman, Robin Ericson, Dorota Drożdz, Amrit Kaur, Sevcan A. Bakkaloglu, Charlotte Samaille, Marsha Lee, Stephanie Tellier, Julia Thumfart, Marc Fila, Bradley A. Warady, Franz Schaefer, Claus P. Schmitt

**Affiliations:** 1 Department for Pediatrics, Nephrology and Hypertension, Medical University of Gdansk, Gdansk, Poland; 2 UCL Great Ormond Street Hospital and Institute of Child Health, London, UK; 3 Hôpital Femme Mère Enfant, Hospices Civils de Lyon, Lyon, France; 4 Children’s Hospital of Fudan University, Shanghai, China; 5 Fondazione IRCCS Ca’ Granda Ospedale Maggiore Policlinico, Milan, Italy; 6 King Abdulaziz University Hospital, King Abdulaziz University, Jeddah, Saudi Arabia; 7 Department of Pediatrics, Seoul National University Children’s Hospital, Seoul National University College of Medicine, Republic of Korea; 8 Aljalila Children’s Specialty Hospital, Department of Pediatric Nephrology, Dubai, United Arab Emirates; 9 Cayetano Heredia Hospital, Lima, Peru; 10 Iran University of Medical Sciences, Tehran, Iran; 11 Starship Children’s Hospital, Auckland, New Zealand; 12 Jagellonian University Medical College, Kraków, Poland; 13 Royal Manchester Children’s Hospital, Manchester, UK; 14 Gazi University Hospital, Ankara, Turkey; 15 Hôpital Jeanne de Flandre, Lille, France; 16 The University of California, San Francisco, California, USA; 17 Dialyse Pediatricue CHU, Toulouse, France; 18 Department of Pediatric Gastroenterology, Nephrology and Metabolic Diseases, Charité Universitätsmedizin Berlin, Germany; 19 Department of Pediatric Nephrology, CHU de Montpellier, Montpellier, France; 20 Children’s Mercy Kansas City, Kansas City, Missouri, USA; 21 Centre for Pediatric and Adolescent Medicine, University of Heidelberg, Germany

**Keywords:** blood pressure, hemodialysis, left ventricular hypertrophy

## Abstract

**Introduction:**

Fluid and salt overload in patients on dialysis result in high blood pressure (BP), left ventricular hypertrophy (LVH) and hemodynamic instability, resulting in cardiovascular morbidity.

**Methods:**

Analysis of 910 pediatric patients on maintenance hemodialysis/hemodiafiltration (HD/HDF), prospectively followed-up with 2758 observations recorded every 6-months in the International Pediatric Hemodialysis Network (IPHN).

**Results:**

Uncontrolled hypertension was present in 55% of observations, with 27% of patients exhibiting persistently elevated predialysis BP. Systolic and diastolic age- and height-standardized BP (BP-SDS) were independently associated with the number of antihypertensive medications (odds ratio [OR] = 1.47, 95% confidence interval 1.39–1.56, 1.36 [1.23–1.36]) and interdialytic weight gain (IDWG; 1.19 [1.14–1.22], 1.09 [1.06–1.11]; all *P* < 0.0001). IDWG was related to urine output (OR = 0.27 [0.23–0.32]) and dialysate sodium (dNa; 1.06 [1.01–1.10]; all *P* < 0.0001). The prevalence of masked hypertension was 24%, and HD versus HDF use was an independent risk factor of elevated age- and height-standardized mean arterial pressure (MAP-SDS) (OR = 2.28 [1.18–4.41], *P* = 0.01). Of the 1135 echocardiograms, 51% demonstrated LVH. Modifiable risk factors included predialysis systolic BP-SDS (OR = 1.06 [1.04–1.09], *P* < 0.0001), blood hemoglobin (0.97 [0.95–0.99], *P* = 0.004), HD versus HDF modality (1.09 [1.02–1.18], *P* = 0.01), and IDWG (1.02 [1.02–1.03], *P* = 0.04). In addition, HD modality increased the risk of LVH progression (OR = 1.23 [1.03–1.48], *P* = 0.02). Intradialytic hypotension (IDH) was prevalent in patients progressing to LVH and independently associated with predialysis BP-SDS below 25th percentile, lower number of antihypertensives, HD versus HDF modality, ultrafiltration (UF) rate, and urine output, but not with dNa.

**Conclusion:**

Uncontrolled hypertension and LVH are common in pediatric HD, despite intense pharmacologic therapy. The outcome may improve with use of HDF, and superior anemia and IDWG control; the latter via lowering dNa, without increasing the risk of IDH.

## Graphical abstract

**Figure:**
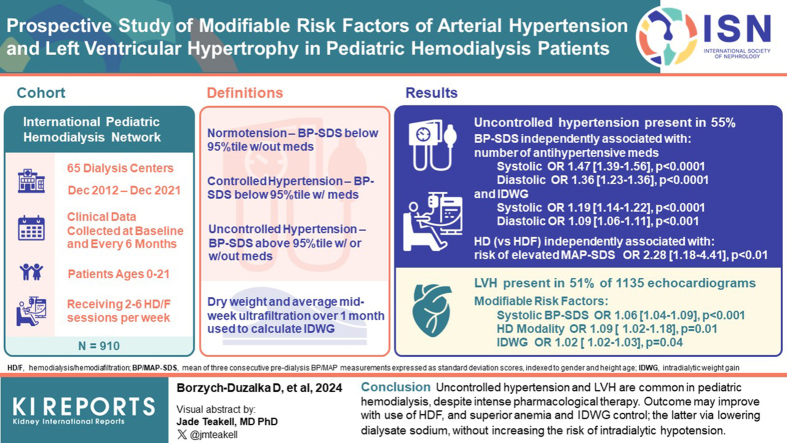

See Commentary on Page 1577

Children and adults with chronic kidney disease have a high risk of cardiovascular disease mortality and morbidity. In dialysis patients, cardiovascular disease mortality risk is greater than 40 times the risk of the age-matched general population and is the leading cause of death.^1^, ^2^, ^3^, ^4^, ^5^ The etiology is multifactorial, consisting of classical risk factors, as well as chronic kidney disease and dialysis related factors. The latter are chronic kidney disease-associated mineral bone disease, anemia, inflammation, and oxidative stress, but also volume and salt overload that are causally associated with arterial hypertension.^6^ Persistent salt and fluid overload and high BP result in end organ damage such as LVH and contribute to poor patient outcome. In patients on HD, fluid overload necessitates high UF rates, which may induce cardiac stunning, reduce renal perfusion, and enhance thirst, which further drives excessive fluid intake.^7^ Treatment should be targeted to break this vicious circle. Therefore, it is essential to understand factors involved in inadequate fluid, salt, and BP control in clinical practice.

The objective of our investigation was to analyze the impact of potentially modifiable risk factors of hypertension and LVH in the largest pediatric patient cohort on maintenance HD to date, prospectively followed-up in great detail by the IPHN registry.

## Methods

### Data Collection and Study Design

The IPHN collects prospective information online on patients on maintenance HD or HDF treated in pediatric dialysis units around the globe (www.pedpd.org). At the time of enrollment, patient demographics, underlying kidney disease, clinical data, and information regarding vascular access are captured. Detailed follow-up data are collected at baseline and every 6 months following HD initiation for all incident and prevalent patients (Supplementary Methods). Data entries are automatically checked for plausibility and completeness. The registry protocol was approved by institutional review boards as required at each participating center. Written parental consent, and when appropriate, assent from patients were obtained. A total of 1285 patients on maintenance HD or HDF treated at 65 pediatric dialysis units in 30 countries were entered in the IPHN database between December 2012 and December 2021. Of these, 243 patients were excluded from the current analysis due to core data incompleteness, having >2 or >6 HD of HDF sessions per week, or as a result of being older than 21 years at registry entry. In addition, 132 patients were excluded due to HD or HDF duration being shorter than 30 days.

### Definitions and Calculations

Predialysis office BP values were the mean of 3 consecutive predialysis midsession measurements, recorded at the first data entry and at each 6-month update. BP data were expressed as SD scores, indexed to gender and height age.^8^ Normotension was defined as predialysis systolic and diastolic BP-SDS below the 95th percentile,^9^ without the use of antihypertensive medication. Controlled hypertension was BP-SDS below the 95th percentile while on antihypertensives therapy, whereas uncontrolled hypertension was defined as elevated BP with or without antihypertensives. Urine output and midweek dialysis session UF were normalized for body surface area (BSA) and body weight. “Dry” weight and average UF during the midweek dialysis session over the prior month was used to calculate relative IDWG (% above dry weight). Ambulatory BP monitoring (ABPM) data were recorded in patients taller than 120 cm and expressed as mean 24-hour, daytime, and nighttime arterial pressure (i.e., MAP-SDS).^8^ Masked hypertension was defined as normal predialysis BP-SDS and 24-hour MAP-SDS above 95th percentile. Echocardiographic data were evaluated according to the guidelines of the American Society of Echocardiography.^10^ Left ventricular mass (LVM) was calculated according to the Devereux formula:^11^

$$LVM\left(g\right)=0.8\times \left(1.04\left[{\left\{LVEDD+PWT+IVST\right\}}^{3}+{\left\{LVEDD\right\}}^{3}\right]+0.6\right)$$

LVM was indexed for the power of its allometric or growth relation with height (height in m^2.7^).^12^ Relative wall thickness, RWT = PWT + IVST/LVEDD and normalized to height age.^12^ LVH was defined as indexed LVM (LVMI) exceeding the 95th percentile for gender and height age.^13^ Eccentric LVH was defined as the presence of LVH along with relative wall thickness below 0.42. Progression to LVH was defined as any increase of LVMI in children aged >8 years and ≥5% increase during follow-up in older children, concomitantly expressing LVH at the last observation. IDH was defined as symptomatic BP decrease requiring medical intervention during HD or HDF session.

### Statistical Analyses

Continuous variables were checked for normal distribution using the Kolmogorov-Smirnoff test and expressed as mean ± SD for normally distributed variables and median and interquartile range (IQR) for nonnormally distributed variables. Categorical variables were expressed as frequency and percentage. Differences in proportions were assessed using χ^2^ test. Associations were determined using Spearman correlation coefficient (*r*). Parameters included in the analyses were age, underlying kidney disease (congenital anomalies of the kidneys and urinary tract vs. glomerulonephritis and others), HD versus HDF modality, vascular access, HD or HDF duration, HD or HDF frequency, total weekly dialysis time, dNa, dialysate calcium, UF rate, small-molecule clearances (Kt/V_urea_), use of body volume monitoring, urine output, serum hemoglobin, albumin, calcium, inorganic phosphate, parathormone, number and type of antihypertensives (renin-angiotensin system antagonists, calcium-channel blockers, beta blockers, and diuretics).

Parameters with *P* < 0.15 in univariable analysis were selected for multivariable analysis. Standardized UF rate and relative IDWG were used interchangeably in all multivariable analyses due to their reciprocal interdependency. Mixed linear model analyses were applied to identify factors associated with changes in predialysis BP-SDS, IDWG, 24-hour MAP-SDS, presence of LVH. Generalized linear model assuming an underlying Poisson distribution and a log-link function was used to assess variables associated with weekly IDH frequency. To account for repeated observations per individual, both univariable and multivariable analyses were weighted for observation number per patient. Region of residence was modeled as the random intercept. For the longitudinal analysis, time-integrated patient-specific mean values were calculated for each variable according to individual observation times. Because children who received HDF were older, for HD versus HDF comparison, all analyses were repeated for age-matched cohort. Due to the low number of patients originating from Latin America and New Zealand, these were excluded from the mixed model analysis. Differences with *P* < 0.05 were considered significant. Data were analyzed using SAS, version 9.4 (SAS Institute).

## Results

### Demographics

Nine hundred ten patients (507 boys; 56%) aged 0 to 21 (median 12.1, IQR 19.0-15.9) years on maintenance HD or HDF at registry entry were included. Patients on HDF were significantly older, more commonly had arteriovenous fistula versus central venous line as vascular access and were less likely to be oligoanuric at study entry. In addition, 84% of these come from Western European centers. Detailed patient characteristics by modality at study entry are presented in Table 1. In 304 patients (33%), only 1 observation was available, whereas 606 (67%) were followed-up with from 6 to 92 months with median follow-up time of 17 (IQR, 11–27) months and 3 (IQR, 2–5) observations per patient. In 582 patients, HD or HDF was terminated during the study, mostly due to kidney transplantation (386 patients, 66%) and transfer to other centers (101 patients, 17%). Twenty-two patients (2.4%) died due to cardiac (*n* = 4), neurologic or pulmonary disease, sepsis, therapy withdrawal (*n* = 3, each), and unknown, sudden death, and accident (*n* = 6).

**Table 1:** Patient characteristics at registry entry according to dialysis modality

Variables	All (*N* = 910)	HD (*n* = 682)	HDF (*n* = 228)	*P*-value
Age (yr), median (IQR)	13.5 (9.3–16.2)	12.9 (7.8–15.9)	14.4 (10.8–16.5)	<0.0001
Gender (male), *n* (%)	507 (55)	377 (55)	130 (57)	0.84
Incident pt., *n* (%)	478 (52)	330 (48)	148 (64)	0.01
Patients with no follow-up data, *n* (%)	210 (23)	164 (24)	46 (20)	0.23
Previous dialysis, *n* (%)	274 (30)	208 (30)	66 (28)	0.67
Previous transplant, *n* (%)	131 (14)	91 (13)	40 (17)	0.03
CAKUT, *n* (%)	377 (42)	257(37)	120 (53)	0.001
Glomerulonephritis	234 (26)	188 (28)	47 (21)	
Other	299 (33)	237 (35)	61 (26)	
Vascular access (CVL), *n* (%)	621 (68)	522 (77)	99 (44)	<0.0001
Oligoanuria, *n* (%)	441 (48)	368 (54)	73 (32)	<0.0001
HD vintage (yr), median (IQR)	1.05 (0.21–1.03)	1.08 (0.22–1.04)	0.93 (0.21–0.78)	0.56
Follow-up time (yr), median (IQR)	0.6 (0.0–0.92)	0.59 (0.0–0.91)	0.9 (0.0–0.96)	0.52
Region, *n* (%)				<0.0001
Western Europe	451 (50)	260 (58)	191 (42)	
Turkey/Middle East	168 (18)	161 (96)	7 (4)	
Asia	123 (14)	121 (98)	2 (2)	
Central Europe	84 (9)	63 (75)	21 (25)	
North America	56 (6)	51 (90)	6 (10)	
Latin America	19 (2)	19 (100)	0 (0)	
New Zealand	8 (1)	7 (88)	1 (12)	

CAKUT, congenital abnormalities of the kidney and urinary tract; CVL, central venous line; HD, hemodialysis; HDF, hemodiafiltration; IQR, interquartile range; pt, patient.

### Dialysis Characteristics

A total of 2758 6-monthly observations were available. Conventional HD was prevalent in 1965 (71%) and HDF in 793 (29%) observations. HDF was performed in postdilution mode in 86% of observations with a median convective flow of 12.8 l/m^2^ BSA (IQR, 11–15), and predilution mode in 14% with a convective flow of 22.7 l/m^2^ BSA (IQR, 15.6–27.3). The median weekly dialysis time was 11.7 (range, 6–26) hours, dialysis frequency was 2 to 6 per week (78% thrice weekly). Blood volume monitoring was reported in 386 observations (14%) (median age, 15.6; range, 2.7–23 years) in 17 centers, and dialysis sodium profiling in 161 observations (5%) in 20 centers. Of the patients, 98% were treated with dialysis machines allowing for modification of dNA concentration.

### Predialysis BP

Antihypertensives were administrated in 62% of observations. The mean number of drugs per observation was 2.1 ± 1.0 (range, 1–5). Calcium-channel blockers were most frequently used (72%), followed by angiotensin-converting enzyme inhibitors or angiotensin 2 receptor blockers (57%), beta blockers (45%), then and diuretics (17%). Monotherapy was prevalent in 567 (21%) and 3 or more antihypertensives in 553 (20%) of observations. Calcium-channel blockers were the mainstays of monotherapy (45%), followed by angiotensin-converting enzyme inhibitors or angiotensin 2 receptor blockers (33%), and beta blockers and diuretics (both 11%). When comparing predialysis BP in patients on monotherapy, the lowest BP was observed with beta blockers (mean systolic BP-SDS, 1.12 ± 1.64) followed by angiotensin-converting enzyme inhibitors or angiotensin 2 receptor blockers (systolic BP-SDS, 1.72 ± 1.49), diuretics (systolic BP-SDS, 1.72 ± 1.68) and calcium-channel blockers (systolic BP-SDS, 1.97 ± 1.27; *P* = 0.0005).

In multivariate approach limited to monotherapy patients, after correcting for age, underlying kidney disease, modality therapy duration, IDWG, serum parathyroid hormone, albumin, and blood hemoglobin, the effect of treatment choice remained significant. Urine output was higher (0.51 vs. 0.26 l/m^2^ BSA per 24 h; *P* < 0.0001) and IDWG lower in patients with diuretic versus other monotherapies (3.1% vs. 3.7%; *P* = 0.04).

The number of antihypertensives decreased with dialysis vintage (*r* = −0.65, *P* = 0.0006) and increased with systolic and diastolic BP-SDS (*r* = 0.34, *r* = 0.30; both *P* < 0.0001). Number of antihypertensives was higher in patients on HD than in those on HDF (1.8 ± 1.32 vs. 1.1 ± 1.12, and 1.6 ± 1.3 vs. 1.0 ± 1.1 in age-matched cohorts; both *P* < 0.0001), without differences in the type of antihypertensives used.

Hypertension was uncontrolled in 1509 observations (55%); in 1102 of these observations (73%), children were on 2.23 ± 1.04 antihypertensives. Hypertension was well-controlled on 1.84 ± 0.92 (*P* < 0.0001 vs. uncontrolled) antihypertensives in 595 (21%) observations. Normotension was present in 654 (24%) of observations. Out of 606 patients with longitudinal follow-up (median 12; IQR, 9–22 months), 168 patients (27%) demonstrated uncontrolled hypertension.

**Figure 1:**
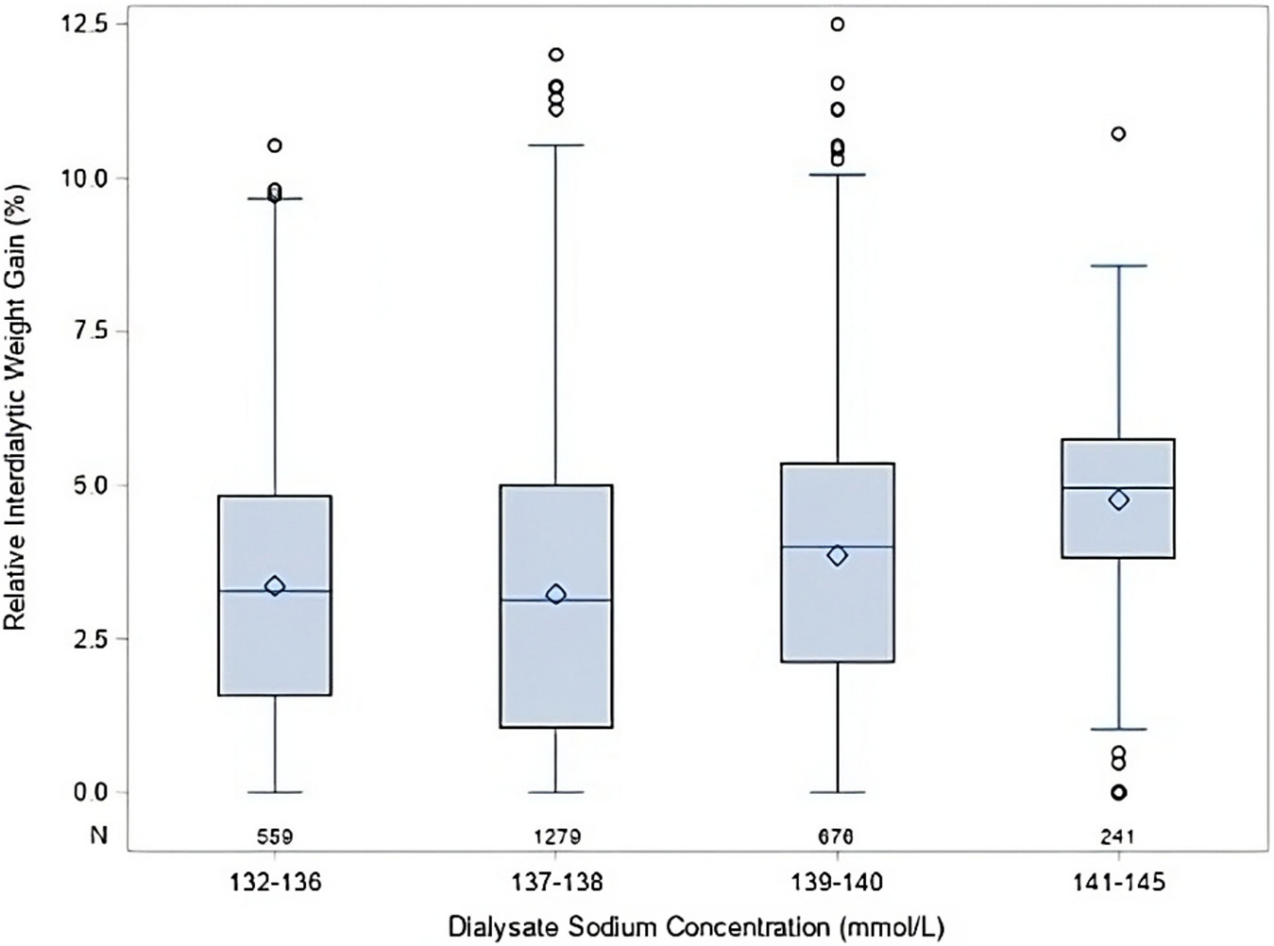
Relative interdialytic weight gain by dialysate sodium concentrations.

**Figure 2:**
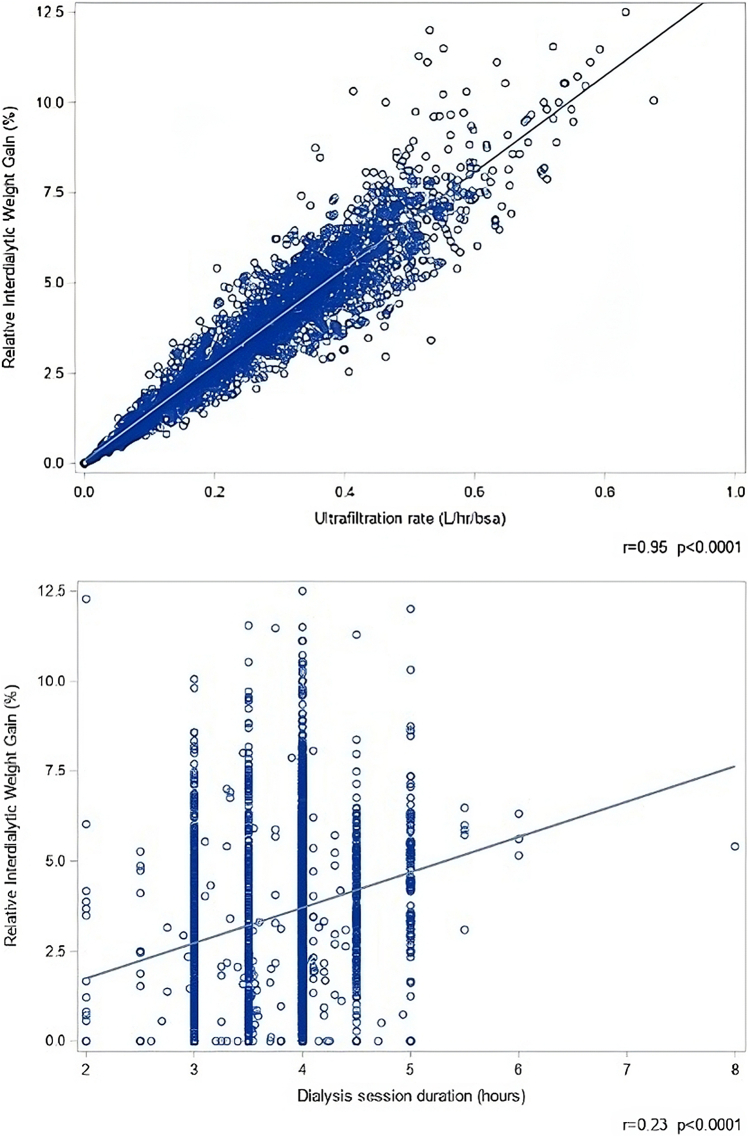
Ultrafiltration rate (upper graph) and dialysis duration (lower graph) versus relative interdialytic weight gain.

Systolic and diastolic BP-SDS were higher in patients with glomerulonephritis than in those with congenital abnormalities of kidney and urinary tract (systolic 1.98 ± 1.65 vs. 1.51 ± 1.78 and diastolic 1.42 ± 1.38 vs. 0.92 ± 1.34; both *P* < 0.0001). Higher systolic and diastolic BP-SDS were associated with HD modality (systolic 1.9 ± 1.04 vs. 1.45 ± 0.81, diastolic 1.32 ± 0.89 vs. 0.84 ± 0.62 in HD vs. HDF; both *P* < 0.0001) and central venous line versus arteriovenous fistula usage (systolic 1.9 ± 1.1 vs. 1.5 ± 0.8 and diastolic 1.3 ± 0.9 vs. 0.9 ± 0.6; both *P* < 0.0001). Associations between BP and clinical or biochemical characteristics is presented in Supplementary Table S1 and correlation of BP-SDS with IDWG in Supplementary Figure S1. In multivariable analysis, higher number of antihypertensives, younger age, underlying disease other than congenital abnormalities of the kidney and urinary tract, and higher relative IDWG were independently associated with higher systolic and diastolic BP-SDS (Table 2). Each 1% increase in relative IDWG was associated with a 19% increase in systolic and a 9% increase in diastolic BP-SDS. In turn, higher IDWG was independently associated with urine output and dNa (Table 3). The distribution of IDWG by dNa is shown in Figure 1, and the correlation of IDWG with UF rate (*r* = 0.95, *P* < 0.0001) and dialysis session duration (*r* = 0.21, *P* < 0.0001) in Figure 2. BP-SDS and IDWG were higher in patients from Asia and Turkey or Middle East.

**Table 2:** General mixed model analysis of factors associated with systolic and diastolic BP-SDS

	Systolic BP-SDS		Diastolic BP-SDS	
Variables	OR (95% CI)	*P*-value	OR (95% CI)	*P*-value
Age (yr)	0.92 (0.91–0.94)	<0.0001	0.94 (0.92–0.96)	<0.0001
IDWG (% body weight)	1.19 (1.14–1.22)	<0.0001	1.09 (1.06–1.11)	<0.0001
Number of AHT drugs	1.47 (1.39–1.56)	<0.0001	1.29 (1.23–1.36)	<0.0001
Primary kidney disease (ref = CAKUT)				
Glomerulonephritis	1.30 (1.03–1.63)	0.02	1.21 (0.99–1.48)	0.06
Other	1.29 (1.04–1.61)	0.02	1.19 (0.96–1.46)	0.09
Dialysis vintage (yr)	0.95 (0.91–0.99)	0.03	0.98 (0.95–1.02)	0.42
Dialysate calcium (mmol/l)	1.26 (0.90–1.76)	0.14	0.97 (0.73–1.28)	0.82
Serum parathyroid hormone (log, pg/ml)	1.03 (0.98–1.09)	0.16	0.98 (0.94–1.04)	0.41
HD modality (vs. HDF)	1.11 (0.92–1.32)	0.26	0.94 (0.85–1.04)	0.21
Dialysate sodium (mmol/l)	0.98 (0.94–1.02)	0.40	0.97 (0.94–1.00)	0.10
Access (CVL vs. AVF)	0.93 (0.77–1.13)	0.51	0.89 (0.81–1.01)	0.05
Albumin (g/l)	0.99 (0.98–1.01)	0.67	0.99 (0.98–1.00)	0.08
Urine output (l/m^2^BSA)	1.02 (0.87–1.21)	0.77	0.96 (0.84–1.09)	0.74
Region (ref = Western Europe)				
Central Europe	1.06 (0.75–1.51)	0.41	1.27 (0.94–1.72)	0.11
North America	0.76 (0.45–1.28)	0.25	0.94 (0.61–1.47)	0.80
Asia	0.98 (0.71–1.36)	0.92	2.49 (1.89–3.28)	<0.001
Turkey/Middle East	1.45 (1.09–1.93)	0.01	1.83 (1.43–2.34)	<0.001

AHT, antihypertension; AVF, arteriovenous fistula; BP-SDS, standard deviation score of blood pressure; CAKUT, congenital abnormalities of the kidney and urinary tract; CI, confidence interval; CVL, central venous line; HD, hemodialysis; HDF, hemodiafiltration; IDWG, interdialytic weight gain; ref, reference.

For variables with units given in parentheses, odds ratios refer to change in likelihood per unit change (e.g., an odds ratio of 1.26 indicates a 26% increase per 1 mmol/l dialysate calcium).

**Table 3:** General mixed model analysis of factors associated with IDWG (% above body dry weight)

	IDWG	
Variables	Odds Ratio (95% CI)	*P*-value
Urine output (l/m^2^ BSA)	0.27 (0.23–0.32)	<0.0001
Dialysate sodium (mmol/l)	1.06 (1.01–1.10)	<0.0001
Dialysis vintage (yr)	1.05 (1.01–1.09)	0.01
Diuretic use	0.71 (0.5–1.06)	0.07
HD modality (vs. HDF)	0.90 (0.77–1.06)	0.18
Region (ref = Western Europe)		
Central Europe	1.28 (0.94–1.72)	0.12
North America	1.42 (0.96–2.10)	0.80
Asia	2.49 (1.89–3.28)	<0.0001
Turkey/Middle East	1.83 (1.44–2.34)	<0.0001

CI, confidence interval; IDWG, interdialytic weight gain; HD, hemodialysis; HDF, hemodiafiltration; ref, reference.

For variables with units given in parentheses, odds ratios refer to change in likelihood per unit change (e.g., an odds ratio of 0.27 indicates a 73% decrease per 1 l/m^2^/d urine output).

### 24-Hour Ambulatory BP

Three hundred eighty-one ABPM recordings were available in 214 patients. Of these, 253 ABPM (66%) in 173 patients (81%) demonstrated uncontrolled hypertension, including 38 observations (15%) with isolated nighttime hypertension. In 92 cases with predialysis office BP below 95th percentile, 24-hour MAP-SDS was elevated, indicating 24% of masked hypertension.

Higher 24-hour MAP-SDS was associated with HD versus HDF modality (2.36 vs. 1.48; *P* = 0.0003), age (*r* = −0.12; *P* = 0.01), Kt/V (*r* = −0.13; *P* = 0.01), and IDWG (*r* = 0.12; *P* = 0.01). Daytime and nighttime MAP-SDS were also lower in patients on HDF (daytime: 1.76 vs. 1.08, *P* = 0.003; nighttime: 2.47 vs. 1.81; *P* = 0.001).

In generalized mixed model analysis, only HD modality (OR = 2.28; 95% confidence interval, 1.18–4.41; *P* = 0.01) remained a significant risk factor for elevated 24-hour MAP-SDS.

### LVH

In 1135 echocardiography studies recorded in 552 patients, 579 (51%) demonstrated LVH, most commonly (61%) with eccentric geometry. In univariable analyses, the presence of LVH was associated with higher systolic and diastolic BP-SDS (2.22 vs. 1.22 and 1.49 vs. 0.82; both *P* < 0.0001), higher IDWG (3.8% vs. 2.8% *P* < 0.0001), lower hemoglobin (10.6 vs. 11.2; *P* < 0.0001), higher serum phosphate (1.84 vs. 1.66; *P* < 0.0001), lower urine output (274 vs. 373 ml/m^2^/d; *P* = 0.001), higher serum parathyroid hormone (426 vs. 315 pg/ml; *P* = 0.0001), and lower albumin (39.3 vs. 40.1g/l; *P* = 0.005). Patients with LVH were more commonly on HD vs. HDF (56% vs. 38%, *P* < 0.0001) and experienced more IDH episodes (0.82/mo vs. 0.63/mo; *P* = 0.06). Dialysis vintage and weekly dialysis time were not different between patients with LVH and those without LVH.

In multivariable analysis, LVH was associated with higher systolic BP-SDS, lower serum hemoglobin, HD versus HDF modality, older age, and higher IDWG, without significant regional variation (Table 4). When replacing relative IDWG by UF/h/m^2^ BSA, which were closely related (*r* = 0.95; *P* < 0.0001), the latter also increased risk of LVH (OR = 1.29; 95% confidence interval, 1.01–1.65; *P* = 0.03). In patients with LVH, median UF/h/m^2^ BSA was 281 (171–384) ml/m^2^/h and 9.8 (5.7–13.1) ml/kg/h; whereas in children without LVH it was 203 (66–314) ml/m^2^/h and 6.9 (2.4–10.4) ml/kg/h (both *P* < 0.0001). In 41% of observations with LVH, the UF rates exceeded 10 ml/kg/h.

**Table 4:** General mixed model analysis of LVH risk factors based on 1135 observations in 552 patients and progression to LVH in 222 patients with longitudinal data available

	LVH		Progression to LVH	
Variable^a^	Odds ratio (95% CI)	*P-*value	Odds ratio (95% CI)	*P*-value
Systolic BP-SDS	1.06 (1.04–1.09)	<0.0001	1.02 (0.97–1.07)	0.39
Blood hemoglobin (g/dl)	0.97 (0.95–0.99)	0.004	1.01 (0.96–1.07)	0.66
Age (yr)	1.02 (1.01–1.04)	0.008	1.02 (1.01–1.04)	0.006
HD modality (ref = HDF)	1.09 (1.02–1.18)	0.01	1.23 (1.03–1.48)	0.02
IDWG (% body weight)	1.02 (1.01–1.03)	0.04	1.02 (0.95–1.05)	0.67
Diastolic BP-SDS	1.02 (0.99–1.05)	0.07	—	—
Kt/V	0.93 (0.88–1.02)	0.08	—	—
Serum phosphate (mmol/l)	1.04 (0.98–1.10)	0.24	—	—
Serum parathyroid hormone (log pg/ml)	1.01 (0.99–1.02)	0.28	1.04 (0.99–1.14)	0.25
Weekly rate of IDH episodes	0.99 (0.98–1.02)	0.63	1.03 (0.97–1.07)	0.34
Urine output (l/m^2^ BSA)	0.97 (0.91–1.04)	0.64	0.98 (0.96–1.02)	0.53
Serum albumin (g/l)	0.99 (0.98–1.01)	0.81	0.98 (0.97–1.02)	0.03
Region (ref = Western Europe)				
Central Europe	0.89 (0.79–1.00)	0.06	0.95 (0.76–1.18)	0.90
North America	1.00 (0.86–1.17)	0.94	1.02 (0.86–1.62)	0.28
Asia	0.97 (0.88–1.07)	0.62	0.79 (0.71–1.06)	0.16
Middle East/Turkey	0.96 (0.86–1.08)	0.52	0.82 (0.64–1.02)	0.07

BP, blood pressure; CI, confidence interval; HD, hemodialysis; HDF, hemodiafiltration, IDWG, interdialytic weight gain; LVH, left ventricular hypertrophy; ref, reference; SDS, SD score.

For variables with units given in parentheses, odds ratios refer to change in likelihood per unit change (e.g., an odds ratio of 0.97 indicates a 3% risk decrease per 1 g/dl blood hemoglobin).

a Time-averaged BP predialysis values and biochemical variables were used as predictors of LVH progression.

Follow-up was available in 222 patients continuing the same dialysis modality (153 HD and 69 HDF) throughout the study for 13 (6–24) months. In 82 of these (68 HD and 14 HDF), LVMI increased and progressed to LVH, most commonly (68%) manifesting with eccentric geometry pattern. Patients who progressed to LVH were older (14.9 vs. 13.1 years, *P* = 0.01) and were commonly on HD (83% vs. 17% on HDF; *P* = 0.0006). Sicty-six percent of patients on HD and 78% of patients on HDF with progression of LVH demonstrated eccentric geometry (*P* = 0.34).

They presented with lower serum albumin (39.3 vs. 40.7; *P* = 0.001), higher frequency of IDH episodes (1.0/mo vs. 0.4/mo; *P* = 0.004) and higher IDWG (4.0% vs. 3.4%; *P* = 0.02). In multivariate analysis, independent risk factors for LVH progression remained older age and HD modality (Table 4).

In multivariable analysis of 304 observations in 139 patients with both echocardiographic and ABPM evaluation, LVH presence (53%) was independently associated with 24-hour MAP-SDS (OR = 1.4; 95% confidence interval, 1.19–1.64; *P* < 0.0001).

### Intradialytic Hypotension

**Figure 3:**
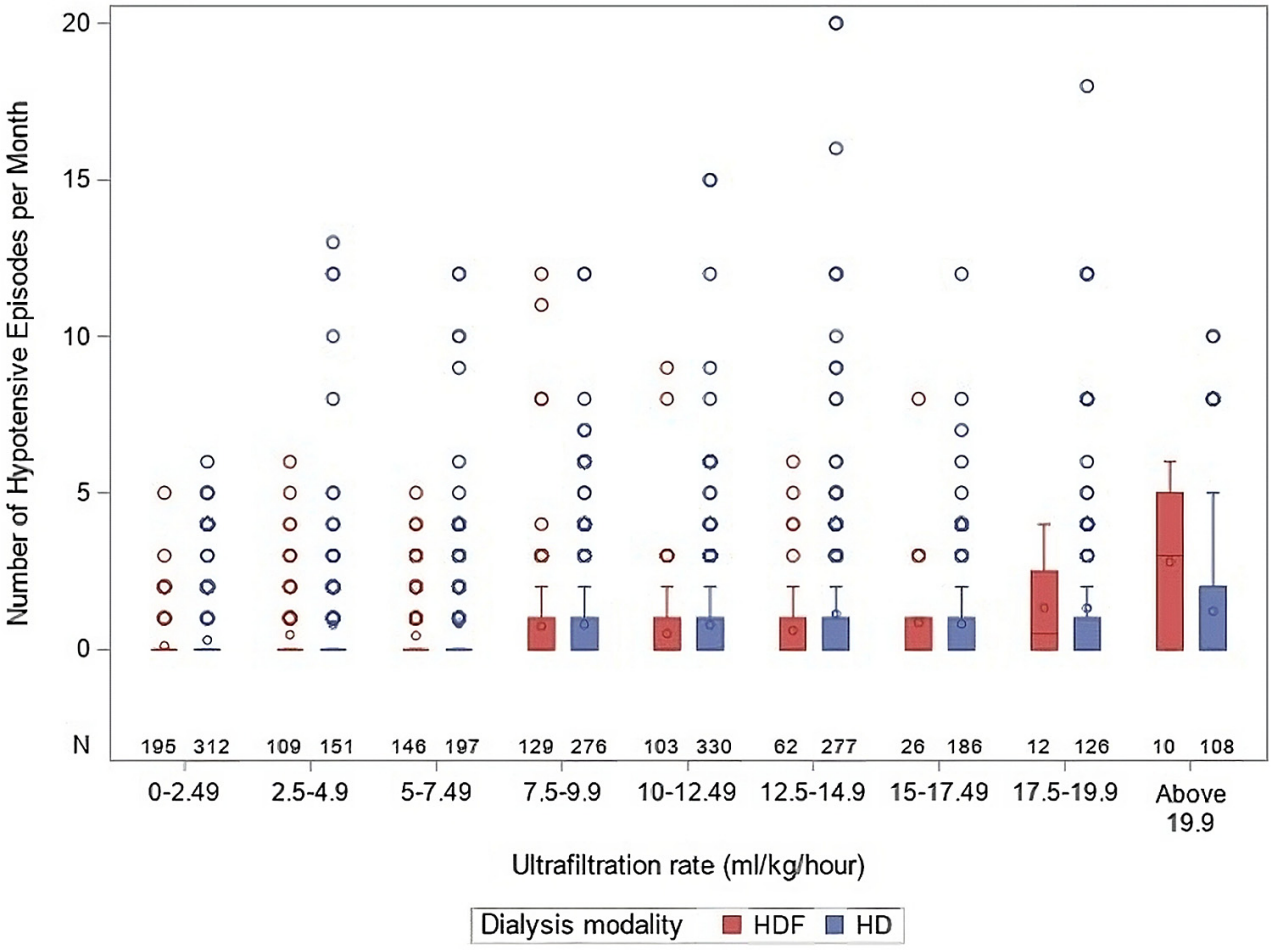
Intradialytic hypotension by ultrafiltration rate and modality. HD, hemodialysis; HDF, hemodiafiltration.

IDH episodes were reported in 20% of 6-monthly updates. The IDH frequency was 0.15 ± 0.43 per week. In the multivariable linear Poisson regression analysis, frequency of weekly IDH was independently predicted by HD versus HDF modality, predialysis office BP below the 25th percentile, serum parathyroid hormone, and UF rate (Figure 3), while inversely by 24-hour urine output, patient age, serum albumin, and number of antihypertensives, but not by dNa (Table 5).

**Table 5:** Univariate and multivariate Poisson regression analysis of factors predicting rate of intradialytic hypotensive episodes

	Univariate		Multivariate	
Variable	RR (95% CI)	*P*-value	RR (95%CI)	*P*-value
Systolic BP below 25th percentile	1.64 (1.40–1.71)	<0.001	1.64 (1.40–1.63)	<0.001
HD modality *(ref = HDF)*	1.68 (1.51–1.88)	<0.001	1.40 (1.22–1.61)	<0.001
Serum parathyroid hormone (log, pg/ml)	1.21 (1.16–1.25)	<0.001	1.12 (1.07–1.15)	<0.001
Ultrafiltration rate (ml/h/kg)	1.06 (1.05–1.07)	<0.001	1.03 (1.02–1.04)	<0.001
Urine output per BSA (l/m^2^)	0.54 (0.47–0.60)	<0.001	0.67 (0.57–0.74)	<0.001
Age (yr)	0.94 (0.93–0.95)	<0.001	0.95 (0.94–0.97)	<0.001
Number of antihypertensives	0.90 (0.87–0.94	<0.001	0.89 (0.85–0.93)	0.001
Dialysis duration (yr)	1.04 (1.02–1.07)	<0.001	0.97 (0.93–0.98)	0.02
Body volume monitoring use	0.43 (0.35–0.52)	<0.001	1.00 (0.81–1.25)	0.63
Dialysate sodium (mmol/l)	1.00 (0.98–1.03)	0.73	—	—
Region *(ref = Western Europe)*				
Central Europe	1.45 (1.34–1.57)	<0.001	1.15 (0.94–1.39)	0.17
North Americal	1.38 (1.25–1.53)	<0.001	1.24 (0.98–1.57)	0.06
Asia	0.17 (0.15–0.20)	<0.001	0.18 (0.14–0.25)	<0.001
Middle East/Turkey	5.44 (5.22–5.68)	<0.001	2.43 (2.12–2.78)	<0.001

BP, blood pressure; CI, confidence interval; HD, hemodialysis; HDF, hemodiafiltration; ref, reference; RR, rate ratios.

For variables with units given in parentheses, RR refer to change in likelihood per unit change (e.g., an RR of 1.06 indicates a 6% risk increase per 1ml/h/kg ultrafiltration rate).

## Discussion

Our study in 910 pediatric patients of HD or HDF followed-up with by the IPHN from 2012 to 2021, demonstrated a 55% prevalence of uncontrolled hypertension, despite intensive antihypertensive therapy. Similar findings were previously reported by the NAPRTCS registry in children initiating dialysis between 1992 and 2004, and by the ESPN/ERA-EDTA registry in children dialyzed between 1999 and 2009; therefore, no improvement has been achieved.^2^^,^^14^ The number of antihypertensives was closely correlated with higher systolic and diastolic BP-SDS, with 3 to 5 antihypertensives used in 20% of the observations. This indicates an inadequate dialytic control of fluid and salt homeostasis.

In patients on antihypertensive monotherapy, BP control was superior with beta blockers, which is in line with findings in adult HD randomized controlled trials.^15^ Diuretic use was associated with both higher urine output and lower IDWG. Risk factors of higher systolic and diastolic BP-SDS were younger age, higher IDWG, primary kidney disease other than congenital abnormalities of the kidney and urinary tract and living in Asia and Turkey/Middle East. Whereas age, primary kidney disease, and region of residence are nonmodifiable factors, IDWG should be potentially adjustable. Each 1% increase in relative IDWG was associated with a 19% increase in systolic BP-SDS and a 9% increase in diastolic BP-SDS. The strongest modifiable predictor of IDWG was dNA. Dialysis machines allowed for modification of dNA in 98% of the patients, that is, this tool is readily available for most pediatric patients. In adults, the optimal dNa remains controversial, with high dNa increasing IDWG and BP, and low dNa increasing the risk of IDH.^16^ In our large pediatric cohort, however, lower dNa was not associated with an increased IDH risk. This is in line with a randomized cross-over trial in 15 children, where a dNa of 135 versus 138 mmol/l was associated with a reduction in IDWG without increasing the incidence of symptomatic sessions.^17^

Although in our cohort lower BP values were associated with arteriovenous fistula use, this was not confirmed in multivariate analysis. Arteriovenous fistula creation affects systemic circulation by increasing cardiac contractility and decreasing peripheral resistance,^18^ which should result in BP drop. Twenty-four-hour ABPM measurements demonstrated a prevalence of masked hypertension of 24%, which predisposes to LVH development.^19^^,^^20^ Single predialysis measurements did not reflect the true BP load.^21^ This and previous findings stress the need for 24-hour ABPM assessment in children on dialysis.^22^^,^^23^ The only independent risk factor for elevated 2hour MAP-SDS was HD versus HDF use, which increased the risk by more than 2-fold. This is in line with the largest interventional study in pediatric patients on HD to date, the 3H-trial, in which 24-hour MAP-SDS was significantly higher in those on HD than in those on HDF, and increased during 12 months on HD but not on HDF.^24^^,^^25^ At present, it is unclear whether these BP effects are due to superior toxin removal, reduced systemic inflammation, and/or increased sodium removal with lower sodium concentration in the substitution fluid.^26^

In the same direction as the high prevalence of uncontrolled hypertension, LVH was present in 51% of the observations. Previous studies in pediatric patients on dialysis published in 2001 and 2011, reported an LVH prevalence of 48% to 85%, depending on dialysis modality and the definition of LVH used.^27^, ^28^, ^29^ A recent NAPRTCS analysis of 518 children followed-up from 2013 to 2020 demonstrated LVH prevalence of 67% at dialysis initiation and 40% to 50% during follow-up.^30^ LVH decreases coronary reserve and contributes to a markedly increased risk of cardiovascular mortality, whereas progression of LVH is the strongest predictor of sudden death in young adults on HD.^31^ The rate of cardiovascular arrest is 100-fold increased in patients on dialysis as compared to the general population.^32^ In our analysis, independent risk factors of LVH were high predialysis systolic BP-SDS and MAP-SDS, low hemoglobin, HD versus HDF use, young age, and high IDWG. Progression to LVH was associated with use of the HD versus HDF modality. Most importantly, all factors except age, should be modifiable in the majority of children. In small observational pediatric HD or HDF studies, rigorous control of fluid status and BP improved LVMI.^33^, ^34^, ^35^, ^36^ In the 3H trial, they improved fluid status;^26^ LVMI was higher in patients on HD than in those on HDF; and in children on HDF, it closely correlated with the improved fluid status.^25^

Patients progressing to LVH had significantly more IDH episodes. IDH results in inadequate fluid and solute removal and in myocardial hypoperfusion with regional wall movement abnormalities.^7^^,^^37^ Recurrent IDH eventually leads to LVH and cardiac dysfunction, which may again increase the risk of IDH in a vicious circle.^7^ Independent risk factors of IDH frequency included predialysis systolic BP below the 25^th^ percentile, younger age, lower number of antihypertensives, HD instead of HDF use, low urine output, and high ultrafiltration rates. A putative mechanistic link between high UF rates, IDH, and LVH is suggested by our finding of 30% higher relative UF rates in children with LVH, with 41% of the cases having UF rates above 10 ml/h/kg. In adults, UF rates above 10 ml/h/kg result in IDH, cardiac stunning, and a higher mortality risk.^38^^,^^39^ It can be only speculated that increased IDH rate in patients with lower number of antihypertensives could be associated with lack of cardioprotective effect associated with their use.^40^ HDF improves intradialytic hemodynamic stability compared to HD, possibly related to higher middle molecule clearance improving vascular endothelial function.^41^^,^^42^ In a small pediatric prospective observational study, less IDH was observed after a switch from HD to HDF.^43^

Although the strength of our analysis relates to the robust set of data available from a large pediatric HD cohort, several limitations of the study should be mentioned. Considering that participation in the IPHN registry is voluntary, we cannot entirely exclude selection bias related to the type of centers reporting to the registry or underreporting of some centers. Even though data collection is strictly prospective, it was not possible to standardize the echocardiographic, BP, and laboratory technologies throughout 65 centers. This methodological variability may have limited the sensitivity of identifying correlates of BP and LVM. In addition, due to the low number of patients per center and country, large scale pediatric HD studies only become possible by the contribution of multiple sites around the globe. Therefore, regional differences in HD populations and treatment practices might influence observed outcomes. Global adjustment for region, as performed in this study, is considered appropriate, although some residual confounding cannot be ruled out.

In conclusion, the largest analysis in pediatric patients on HD or HDF, to date, demonstrates that hypertension and LVH, predominant cardiovascular risk factors in patients on dialysis, are still prevalent in the majority of individuals. The intensity of pharmacologic treatment was associated with worse BP control, indicating that inadequate dialytic fluid and salt control is the key underlying mechanism. Our findings provide strong evidence for actions to be taken to help improve cardiovascular outcomes. These include use of HDF instead of HD, and volume control by decreasing IDWG, with the latter probably achievable by reductions in dNa without increasing the risk of IDH.

## Disclosure

All the authors declared no competing interests.

## Supplementary Materials

Supplementary File (PDF) **Supplementary Methods. Figure S1.** Relationship between predialytic systolic (upper graph) and diastolic blood pressure SDS (lower graph) and relative interdialytic weight gain (%) in 2758 six-monthly observations. Systolic and diastolic BP-SDS correlate with relative interdialytic weight gain (*r* = 0.26/0.24; both *P* < 0.0001). **Table S1.** Spearman’s rank-order correlations of diastolic and systolic BP-SDS. **STROBE Statement.**

## Footnotes

Supplementary File (PDF)

**Supplementary Methods.**
**Figure S1.** Relationship between predialytic systolic (upper graph) and diastolic blood pressure SDS (lower graph) and relative interdialytic weight gain (%) in 2758 six-monthly observations. Systolic and diastolic BP-SDS correlate with relative interdialytic weight gain (*r* = 0.26/0.24; both *P* < 0.0001). **Table S1.** Spearman’s rank-order correlations of diastolic and systolic BP-SDS. **STROBE Statement.** The study followed the Strengthening the Reporting of Observational Studies in Epidemiology (STROBE) reporting guidelines.
