# Supplementary material for: Prospective Study of Modifiable Risk Factors of Arterial Hypertension and Left Ventricular Hypertrophy in Pediatric Patients on Hemodialysis
Source: Kidney Int Rep. 2024 Mar 18;9(6):1694–704. doi: 10.1016/j.ekir.2024.03.016 (PMC11184401; doi:10.1016/j.ekir.2024.03.016)
Supplement: Supplementary File (PDF) — Supplementary Methods. Figure S1. Relationship between predialytic systolic (upper graph) and diastolic blood pressure SDS (lower graph) and relative interdialytic weight gain (%) in 2758 six-monthly observations. Systolic and diastolic BP-SDS correlate with relative interdialytic weight gain (r = 0.26/0.24; both P < 0.0001). Table S1. Spearman’s rank-order correlations of diastolic and systolic BP-SDS. STROBE Statement. [file mmc1.pdf]

Supplemental methods:

6 monthly follow-up data comprise dialysis modality characteristics, pre-dialysis mid-week blood pressure (mean of three measurements), euvolemic weight, hematology and serum biochemistry results (hemoglobin, ferritin, transferrin saturation, HCO3, serum calcium, phosphate, parathormone, albumin and CRP), residual diuresis, renal and dialytic small-molecule clearances (Kt/Vurea). Dialysis related parameters include dialysis modality, dialysis duration and frequency, blood and dialysate flow, achieved ultrafiltration, dialysate sodium, calcium and buffer, as well as information on blood volume monitoring, ultrafiltration and sodium profiling use. Medication section includes information on number and class of antihypertensives, bicarbonate supplementation, type of phosphate binder, active vit D3 sterol, lipid lowering agent, erythropoietin and growth hormone use. In addition, the results of optional echocardiographic investigations and 24-hour ambulatory BP monitoring (ABPM) are recorded, provided these were performed close to clinic visits (mean time lag -4 ± 8 weeks, not significantly different from 0). In the echocardiogram input section of the registry, left ventricular end-diastolic diameter (LVEDD), posterior wall thickness (PWT), and diastolic interventricular septal thickness (IVST) are collected. The ABPM values include 24h, daytime and nighttime MAP. Hypotensive episodes (defined as significant, symptomatic BP drop, as per clinical judgement) are reported as cumulative number of episodes within the 4 weeks preceding the data entry. Patients are followed up until HD discontinuation, recorded reasons include kidney transplantation (deceased donor or living related), loss to follow-up, transfer to other centers, change modality to peritoneal dialysis , death, recovery of native kidney function, or therapy withdrawal.

Supplemental Tables:

Table S1. Spearman's rank-order correlations with diastolic and systolic BP-SDS

	**Systolic BP-SDS**		**Diastolic BP-SDS**	
**Parameter**	**Correlation coefficient**	**p value**	**Correlation coefficient**	**p value**
Number of AHT	0.34	<0.0001	0.3	<0.0001
Age (years)	-0.23	<0.0001	-0.2	<0.0001
IDWG (% body weight)	0.26	<0.0001	0.24	<0.0001
Blood hemoglobin (g/dl)	-0.1	<0.0001	-0.1	<0.0001
Urine output (L/m2 BSA)	-0.1	<0.0001	-0.13	<0.0001
HD/HDF duration (years)	-0.07	<0.0002	-0.07	<0.0002
Serum albumin (g/L)	-0.06	0.001	-0.07	0.003
Parathormone (log, pg/ml)	0.07	0.001	0.05	0.003
Dialysate Calcium (mmol/L)	0.04	0.01	0.03	0.007
Dialysate Sodium (mmol/L)	0.002	0.88	0.06	0.001
KT/V	0.04	0.03	0.01	0.44
Weekly dialysis time (hours)	0.02	0.31	0.02	0.23

Supplemental Figures:

Figure S1.Relationship between pre-dialytic systolic (left graph) and diastolic blood pressure SDS (right graph) and relative interdialytyic weight gain in 2758 six-monthly observations. Systolic and diatsolic BP SDS correlate with relative interdialytic weight gain (r=0.26/0.24, both p<0.0001)

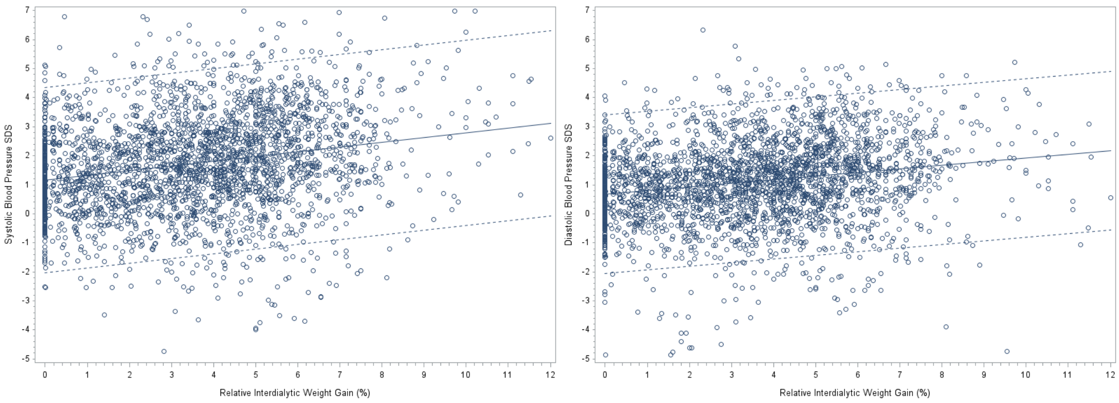

Appendix

The following Principal Investigators are active contributors to the IPHN Registry: **Argentina:** L. Alconcher, Hospital Interzonal General, Bahia Blanca, P.A. Coccia, Hospital Italiano de Buenos Aires, A. Suarez, Hospital de Nińos Sor. Maria Ludovica La Plata, Patricia G. Valles, Hospital Pediatric Humberto Notti, Mendoza; **Canada:** Ch. Licht, Hospital for Sick Children, Toronto; **Chile:** F. Cano, Hospital Luis Calvo Mackenna, Santiago, M.A. Contreras, Roberto del Rio Hospital, Santiago. China: H. Xu, Children's Hospital of Fudan University, Shanghai. **Colombia:** J.J. Vanegas, Instituto del Rinon, Medellin; L.M. Higuita, Baxter Servicio al Cliente Colombia, Medellin.**China:** Yihui Zhai, Children's hospital of fundan University, Shanghai; E. Chan, Princess Margaret Hospital, Hong Kong; **Czech Republic**: K. Vondrak, University Hospital Motol, Prague. **Finland**: J. Lauronen, Hospital for Children and Adolescents, Helsinki**. France**: B. Ranchin, Hôpital Femme Mčre Enfant, Lyon; A.Zaloszyc, Children's Dialysis Center, Strasbourg; Ch. Samaille, Hospital Jeanne de Frandre, Lille; M. Fila, Pediatric Nephrology Unit, Montpellier, I. Vrillon, CHRU, Nancy, S. Tellier,Dialyse Pediatricue CHU, Toulouse.**Germany**: J.Thumfart, Charité Virchow-Klinikum, Berlin; L.Weber University Hospital, Cologne, Cologne; R. Büscher, Children's Hospital Essen; CP. Schmitt, F. Schaefer, D.Borzych-Duzalka Center for Pediatrics and Adolescent Medicine, Heidelberg; G. Klaus, KfH Kidney Center, Marburg;. **Greece:** V. Askiti, A&P Kyriakou Children's Hospital, Athens, F. Papacristou, Aristoteles University, Thesaloniki. **Hungary:** AJ. Szabo, Semmelweis University, Budapest**. India:** N. Kamath, St. John's Medical College, Bangalore, B. Basu, NRS Medical College & Hospital, Kolkata; A. Bagga, All India Institute of Medical Sciences, New Delhi**, Iran:** N. Hooman, Iran University of Medical Sciences, Tehran. **Italy:** F. Paglialonga, S. Testa, Fondazione Ospedale Maggiore Policlinico, Milano; E. Verrina, G. Gaslini Institute, Genova; S E. Vidal, Pediatric Nephrology, Dialysis and Transplant Unit, Padova; G. Leozappa, Department of Nefrologia-Urologia, Roma, **Republic of Korea:** Hee Gyung Kang, Seoul National University Children's Hospital, Seoul; **Malaysia:** YN Lim, Kuala Lumpur Hospital, Kuala Lumpur. **Macedonia**: : E. Sahpazova Pediatric Clinic, Skopje **Nicaragua**: Y. Silva, Hospital Infantil de Nicaragua, Managua. **Oman:** M. Al Ryami, Royal Hospital, Muscat. **New Zealand:** R. Erickson, Starship Children's Hospital, Auckland. **Peru**: R. Loza Munarriz, Cayetano Heredia Hospital, Lima. **Poland:** A.M. Zurowska, D. Borzych-Duzalka, Medical University, Gdansk; D. Drozdz, Jagiellonian University Medical College; M. Szczepanska, Dialysis Division for Children, Zabrze; **Philippines:** A. Marbella, National Kidney and Transplant Institute, Quezon City. **Portugal**: T. Francico, Hospital D. Estefania, Lisboa. **Saudi Arabia**: J. Kari, King Abdul Aziz University Hospital, Jeddah. **Serbia:** D. Kruscic, University Children's Hospital, Belgrade. **Singapore:** H.K. Yap, Shaw-NKF-NUH Children's Kidney Center. **Spain:** G. Ariceta, University Hospital Materno-Infantil Vall d'Hebron, Barcelona. **Sweden:** L. Swartz, Barnkliniken, Lund. **Turkey:** A. Bayazit, Cukrova University, Adana A.S. Bakkaloglu, Hacettepe University, Ankara; S. Bakkaloglu, Gazi University, Ankara; I. Bilge, Department of Pediatric Nephrology, Çapa-Istanbul: Tepecik Children and Research Hospital, Izmir; S. Mir, Ege University Faculty of Medicine, Izmir-Bornova.

United Arab Emirates: E. Simkova, Al Jalila Hospital, Dubai. **United Kingdom**: R. Shroff, Great Ormond Street Hospital, London; C. Ried, Evelina Hospital, London. A. Kaur, Royal Manchester Hospital, Manchaster; **Uruguay:** J. Grünberg, SE.N.NI.AD, Montevideo. **United States:** H. Patel, Children's Hospital, Columbus; B.A. Warady, Children's Mercy Hospital, Kansas City; M. Lee, The University of California, San Francisco; M. Rheault, University of Minnesota, Amplatz Children's Hospital, Minneapolis; M. Pradhan, The Children's Hospital of Philadelphia, Philadelphia; J. Flynn, Seattle Children's Hospital, Seattle; C. Wong, Lucile Packard Children’s Hospital, Palo Alto.

STROBE Statement—checklist of items that should be included in reports of observational studies

	Item No	Recommendation	Page No
**Title and abstract**	1	(*a*) Indicate the study's design with a commonly used term in the title or the abstract	2
		(*b*) Provide in the abstract an informative and balanced summary of what was done and what was found	4
**Introduction**			
Background/rationale	2	Explain the scientific background and rationale for the investigation being reported	6
Objectives	3	State specific objectives, including any prespecified hypotheses	6
**Methods**			
Study design	4	Present key elements of study design early in the paper	7
Setting	5	Describe the setting, locations, and relevant dates, including periods of recruitment, exposure, follow-up, and data collection	7
Participants	6	(*a*) *Cohort study*—Give the eligibility criteria, and the sources and methods of selection of participants. Describe methods of follow-up<br>*Case-control study*—Give the eligibility criteria, and the sources and methods of case ascertainment and control selection. Give the rationale for the choice of cases and controls<br>*Cross-sectional study*—Give the eligibility criteria, and the sources and methods of selection of participants	7
		(*b*) *Cohort study*—For matched studies, give matching criteria and number of exposed and unexposed<br>*Case-control study*—For matched studies, give matching criteria and the number of controls per case	

Variables	7	Clearly define all outcomes, exposures, predictors, potential confounders, and effect modifiers. Give diagnostic criteria, if applicable	7-8
Data sources/ measurement	8*	For each variable of interest, give sources of data and details of methods of assessment (measurement). Describe comparability of assessment methods if there is more than one group	7-8
Bias	9	Describe any efforts to address potential sources of bias	7
Study size	10	Explain how the study size was arrived at	6
Quantitative variables	11	Explain how quantitative variables were handled in the analyses. If applicable, describe which groupings were chosen and why	7
Statistical methods	12	(*a*) Describe all statistical methods, including those used to control for confounding	8-9
		(*b*) Describe any methods used to examine subgroups and interactions	8-9
		(*c*) Explain how missing data were addressed	
		(*d*) *Cohort study*—If applicable, explain how loss to follow-up was addressed<br>*Case-control study*—If applicable, explain how matching of cases and controls was addressed<br>*Cross-sectional study*—If applicable, describe analytical methods taking account of sampling strategy	
		(*e*) Describe any sensitivity analyses	

Continued on next page

Results

Participants	13*	(a) Report numbers of individuals at each stage of study—eg numbers potentially eligible, examined for eligibility, confirmed eligible, included in the study, completing follow-up, and analysed	9-12
		(b) Give reasons for non-participation at each stage	9-12
		(c) Consider use of a flow diagram	
Descriptive data	14*	(a) Give characteristics of study participants (eg demographic, clinical, social) and information on exposures and potential confounders	9
		(b) Indicate number of participants with missing data for each variable of interest	9
		(c) *Cohort study*—Summarise follow-up time (eg, average and total amount)	10-13
Outcome data	15*	*Cohort study*—Report numbers of outcome events or summary measures over time	10-13
		Case-control study—Report numbers in each exposure category, or summary measures of exposure	
		Cross-sectional study—Report numbers of outcome events or summary measures	
Main results	16	(*a*) Give unadjusted estimates and, if applicable, confounder-adjusted estimates and their precision (eg, 95% confidence interval). Make clear which confounders were adjusted for and why they were included	10-13
		(*b*) Report category boundaries when continuous variables were categorized	
		(*c*) If relevant, consider translating estimates of relative risk into absolute risk for a meaningful time period	
Other analyses	17	Report other analyses done—eg analyses of subgroups and interactions, and sensitivity analyses	9

Discussion

Key results	18	Summarise key results with reference to study objectives	14-15

Limitations	19	Discuss limitations of the study, taking into account sources of potential bias or imprecision. Discuss both direction and magnitude of any potential bias	16
Interpretation	20	Give a cautious overall interpretation of results considering objectives, limitations, multiplicity of analyses, results from similar studies, and other relevant evidence	14-16
Generalisability	21	Discuss the generalisability (external validity) of the study results	16
**Other information**			
Funding	22	Give the source of funding and the role of the funders for the present study and, if applicable, for the original study on which the present article is based	

*Give information separately for cases and controls in case-control studies and, if applicable, for exposed and unexposed groups in cohort and cross-sectional studies.

Note: An Explanation and Elaboration article discusses each checklist item and gives methodological background and published examples of transparent reporting. The STROBE checklist is best used in conjunction with this article (freely available on the Web sites of PLoS Medicine at http://www.plosmedicine.org/, Annals of Internal Medicine at http://www.annals.org/, and Epidemiology at http://www.epidem.com/). Information on the STROBE Initiative is available at www.strobe-statement.org.
